# Promoting Interfacial Interactions with the Addition of Lignin in Poly(Lactic Acid) Hybrid Nanocomposites

**DOI:** 10.3390/polym13020272

**Published:** 2021-01-15

**Authors:** Bindu Patanair, Allisson Saiter-Fourcin, Sabu Thomas, Martin George Thomas, Poornima Parathukkamparambil Pundarikashan, Kalaprasad Gopalan Nair, Varsha Krishna Kumar, Hanna J. Maria, Nicolas Delpouve

**Affiliations:** 1 Normandie Univ, UNIROUEN, INSA Rouen, CNRS, GPM, 76000 Rouen, France; bindu_patanair@yahoo.com (B.P.); allison.saiter@univ-rouen.fr (A.S.-F.); 2 School of Chemical Sciences, School of Energy Materials and International and Inter-University Centre for Nanoscience and Nanotechnology, Mahatma Gandhi University, Priyadarshini Hills P.O, Kottayam, Kerala 686 560, India; martinpsrt@gmail.com (M.G.T.); hannavidhu@gmail.com (H.J.M.); 3 Sree Narayana College, Nattika, Affiliated to the University of Calicut, Kerala 680 566, India; poornimapp1995@gmail.com (P.P.P.); gkalaprasad@gmail.com (K.G.N.); 4 Majlis Arts and Science College Puramannur, Malappuram Dist, Kerala 676 552, India; varshakrishnavinz@gmail.com

**Keywords:** PLA, CNT, rGO, MMT, glass transition, crystallization, morphology, thermal stability, calorimetry, cooperativity

## Abstract

In this paper, the calorimetric response of the amorphous phase was examined in hybrid nanocomposites which were prepared thanks to a facile synthetic route, by adding reduced graphene oxide (rGO), Cloisite 30B (C30B), or multiwalled carbon nanotubes (MWCNT) to lignin-filled poly(lactic acid) (PLA). The dispersion of both lignin and nanofillers was successful, according to a field-emission scanning-electron microscopy (FESEM) analysis. Lignin alone essentially acted as a crystallization retardant for PLA, and the nanocomposites shared this feature, except when MWCNT was used as nanofiller. All systems exhibiting a curtailed crystallization also showed better thermal stability than neat PLA, as assessed from thermogravimetric measurements. As a consequence of favorable interactions between the PLA matrix, lignin, and the nanofillers, homogeneous dispersion or exfoliation was assumed in amorphous samples from the increase of the cooperative rearranging region (CRR) size, being even more remarkable when increasing the lignin content. The amorphous nanocomposites showed a signature of successful filler inclusion, since no rigid amorphous fraction (RAF) was reported at the filler/matrix interface. Finally, the nanocomposites were crystallized up to their maximum extent from the glassy state in nonisothermal conditions. Despite similar degrees of crystallinity and RAF, significant variations in the CRR size were observed among samples, revealing different levels of mobility constraining in the amorphous phase, probably linked to a filler-dimension dependence of space filling.

## 1. Introduction

The bio-renewability, ease of processing, and suitability for mechanical recycling [1], as well as decent mechanical properties, make poly(lactic acid) (PLA) among the best substitutes to polymers derived from petroleum sources [2]. However, PLA has certain drawbacks, such as a small elongation at break (<10%), poor impact strength (~5 kJ m^−2^), low heat-deflection temperature (<60 °C), and poor UV-light barrier properties [3]. Blending PLA with either other polymers or nanofillers offers more opportunities to tailor its properties. For instance, PP/PLA-blend nanocomposites with multiwalled carbon nanotubes (MWCNT) [4] have enhanced tensile strength and electrical conductivity. Hybrid nanocomposites of PLA with nanocellulose/nanoclay have enhanced barrier properties and improved thermo-mechanical properties [5]. More specifically, nanocomposites, including nucleophilic nanofillers like nanoclay, graphene oxide (GO), and MWCNT, in PLA exhibit better protection against UV radiation [6], improved Young’s modulus, antibacterial activity, potential use in biomedical applications [7], and increased thermal conductivity, as well as mechanical and electrical properties [8]. 

Lignin, one of the most abundant bio-macromolecules on earth, is biodegradable, nontoxic, and low-cost. Commercially, it is usually easily obtained as a byproduct in the pulp and paper industry. Its use is also regularly reported in biorefineries [9,10,11] and in carbon-fiber manufacturing [12,13]. Moreover, it exhibits interesting mechanical properties and decent thermal resistance. Hence, there is an increased amount of research on the use of lignin as a biomaterial [14,15,16,17,18,19,20,21]. Additionally, lignin has been used as an adhesion promoter in PLA/cotton-fiber composites [22]. The possibility of improving interfacial adhesion thanks to lignin is the consequence of the numerous intermolecular bonds it can be involved in. Therefore, it is possible to associate it in hybrid nanocomposites with PLA via interactions with nanofillers like MWCNT, reduced graphene oxide (rGO), and Cloisite 30B (C30B), which is an organically-modified montmorillonite nanoclay. 

Whilst the nanocomposite morphology has been widely probed by X-ray diffraction and electron microscopy, interest in modulated temperature-differential scanning calorimetry (MT-DSC) has grown these recent years. Several studies have tentatively related the nanocomposite structure to the cooperativity length [23,24,25,26,27,28,29,30]. The method developed by Donth et al. [31,32] in the framework of the cooperative rearranging-regions (CRR) concept, introduced by Adam and Gibbs [33], makes it possible to estimate the cooperativity length from the mean temperature fluctuation associated with the glass transition, which is accessible thanks to MT-DSC. In a previous study on PLA/clay nanocomposites [34], we reported that exfoliated systems exhibit an increase in the CRR size in comparison to neat PLA, in agreement with the observations of Chen et al. on polystyrene/clay nanocomposites [23], whereas intercalation is characterized by a decrease in the CRR size. Calorimetric investigations also provide information regarding the mobility restrictions at the filler/matrix interface. These restrictions are characterized by a decrease of the heat-capacity step at the glass transition, revealing that a part of the amorphous phase does not mobilize. This interphase is named the *rigid amorphous fraction* (*X_RAF filler_*) [35], in reference to the similar behavior commonly reported at the crystal/amorphous interface (*X_RAF crystal_*). It is known that *X_RAF crystal_* strongly affects the macroscopic properties, such as mechanical or barrier properties [36,37]. Klonos et al. [38] showed that *X_RAF filler_* hinders thermal diffusivity, whereas *X_RAF crystal_* facilitates heat transport. 

Thus, we used MT-DSC in this study to characterize PLA/lignin nanocomposites containing nanosheets (rGO), layer-like nanoclays (C30B), or nanotubes (MWCNT). This set of nanofillers was chosen in order to highlight possible shape and size effects influencing both the morphological and thermal properties of the hybrid nanocomposites. Qualitative filler dispersion was assessed from field-emission scanning-electron microscopy (FESEM) and transmission-electron microscopy (TEM). The thermal stability was evaluated from thermogravimetric analysis (TGA). Furthermore, cooperativity and the rigid amorphous fraction were estimated in amorphous and semi-crystalline samples. Since a number of the properties of PLA depend on its degree of crystallinity, the impact of the nanofiller choice on the crystallization kinetic was also reported. 

## 2. Materials and Methods 

### 2.1. Preparation of PLA/Lignin Hybrid Nanocomposites

Lignin was extracted from *Prosopis juliflora* (Sw.), collected from Gujarat, India. PLA-L105, which is named “PLLA” in the present paper, was purchased from Corbion^®^, Amsterdam, Netherlands with *M_W_* = 148 kg mol^−1^, L-enantiomeric purity of 99.5%, and with a melting temperature *T_m_* = 180–200 °C. MWCNT were obtained from nanocyl (NANOCYL^®^ NC7000), Sambreville, Belgium. C30B was made by Southern Clay Products^®^, Gonzales, Texas, USA. rGO was synthesized using a previously reported procedure [39]. Graphite powder, KMnO_4_, NaNO_3_, H_2_SO_4_, and H_2_O_2_ (30%) were obtained from Merck^®^, Mumbai, India.

The components were mixed in chloroform using a probe sonicator. For each composition, 1 wt% of lignin/chloroform mixture and 1 wt% of filler/chloroform (rGO, MWCNT, or C30B) was added into the PLLA/chloroform mixture. The sonicated mixture of PLLA/lignin/nanofiller was cast on a petri dish and dried in an air oven at 70 °C. 

In this paper, the nanocomposites are named PLLA_lignin_*X*, with *X* being the filler. The respective weight percentages of lignin (*y*) and nanofiller (*z*) are indicated as (*y*:*z*). When nothing is mentioned, the ratio between lignin and nanofiller is (1:1).

### 2.2. Field-Emission Scanning-Electron Microscopy (FESEM) and Transmission-Electron Microscopy (TEM)

The fracture-surface morphology was taken at 1 kV with the help of a Zeiss^®^, Marly le Roi, France, Gemini LEO 1530 apparatus at 60 kV. The samples were cryo-fractured and dried, and then the fracture surface was coated with a thin film of carbon (about 20–30 nm thickness) before performing the FESEM analysis. 

TEM micrographs of the samples were taken using a Jeol JEM-2100^®^, Tokyo, Japan transmission-electron microscope with an accelerating voltage of 200 keV. Ultrathin sections of bulk specimens (about 100 nm in thickness) were obtained by crosscutting with an ultramicrotome fitted with a diamond knife.

### 2.3. Thermogravimetric Analysis (TGA)

Thermogravimetric analyses (TGA) were carried out using a TGA Discovery instrument from TA Instruments^®^, Guyancourt, France. The analyses were carried out under nitrogen atmosphere at 25 mL min^−1^ flow rate, in the temperature range of 30–800 °C and a scanning rate of 10 K min^−1^ on 5–10 mg samples. 

### 2.4. Modulated Temperature-Differential Scanning Calorimetry (MT-DSC)

MT-DSC experiments were conducted on a DSC Q100 and DSC Q2000 from TA Instruments^®^, Guyancourt, France, coupled with a refrigerated cooling system. The experiments were conducted under nitrogen atmosphere at 50 mL min^−1^ flow rate. The samples were positioned in Tzero^®^ standard aluminum pans. Baseline correction and cell-capacitance control were done using standard Tzero^®^ technology. To calibrate the temperature and energy, a standard sample of Indium (*T_m_* = 156.60 °C and Δ*H_m_* = 28.38 J g^−1^) was used. The calibration of the specific heat capacity was carried out using sapphire as a reference. Before the calorimetric investigations, the samples (about 5 mg) were placed in a desiccator under P_2_O_5_ and stored under a dehumidified atmosphere for at least one week. 

To compare the crystallization kinetics between materials, the samples were first melted to erase the thermal history and optimize the thermal contact between the sample and the crucible. Then, the samples were cooled to 0 °C at 50 K min^−1^. A heating scan was subsequently performed from 0 °C to 200 °C in heat-only conditions (oscillation amplitude of ±0.318 °C, oscillation period of 60 s, and heating rate of 2 K min^−1^). These conditions prevent cooling in the modulation period, as the instantaneous heating rate is never negative. They are recommended for analyzing crystallization and melting events without the biases caused by melt-recrystallization processes [40]. 

Glass-transition-characteristic parameters and CRR size were obtained by applying heat–cool-modulation parameters (oscillation amplitude of ±2.5 °C, oscillation period of 100 s, and a heating rate equal to 1 K min^−1^) on amorphous and semi-crystalline samples. These conditions respect the minimum number of modulation steps needed to investigate the glass transition, and were previously shown to be efficient for the study of PLA nanocomposites [34]. Amorphization was done using a Perkin Elmer^®^, Villebon-sur-Yvette, France, DSC8500 apparatus. The samples were cooled down from the melt to −50 °C at 300 K min^−1^. Then, a first MT-DSC heat-cool scan was performed from 0 to 105 °C, i.e., until each sample reached its maximum crystallization extent, with the glass transition being recorded at about 60 °C and the cold crystallization proceeding between 80 and 100 °C. In a second step, each sample was cooled to 0 °C at 50 K min^−1^ before being heated again to 105 °C in heat–cool conditions. 

### 2.5. Cooperative Rearranging Regions (CRR)

The complete deconvolution procedure suggested by Reading [40] was applied, giving access to MT-DSC signals, with *C*′ and *C*″ being the in-phase and the out-of-phase components of C*, the complex heat capacity, respectively. More details regarding phase-lag correction and heat-capacity estimation can be found in [41]. The number of relaxing structural units per CRR, *N_α_*, was estimated according to [32]:

(1) $$N_{\alpha }=\frac{N_{A}\;\left(\frac{1}{\mathrm{Cp},{}_{\mathrm{glass}\;\left(T\alpha \right)}}-\frac{1}{\mathrm{Cp},{}_{\mathrm{liquid}\;\left(T\alpha \right)}}\right)\;k_{B}\;T_{\alpha }^{2}\;}{M_{0}\;{\left(\delta T\right)}^{2}}$$

where *T_α_* is the dynamic glass-transition temperature, *k_B_* the Boltzmann constant, Cp the heat capacity at constant pressure, *M*_0_ the molar mass of one structural unit (one repeating unit in the present case, i.e., *M*_0_ = 72 g mol^−1^), *N_A_* the Avogadro number, and *δT* the mean temperature fluctuation related to the glass-transition temperature of an average CRR. *T_α_* is defined as the maximum of *C*″ Gaussian fit when *δT* is its standard deviation. $\mathrm{Cp},{\;}_{\mathrm{glass}\;\left(T\alpha \right)}$ and $\mathrm{Cp},{\;}_{\mathrm{liquid}\;\left(T\alpha \right)}$ are determined by prolonging *C*′ glass and liquid lines to *T_α_*.

## 3. Results and Discussion

**Figure 1:**
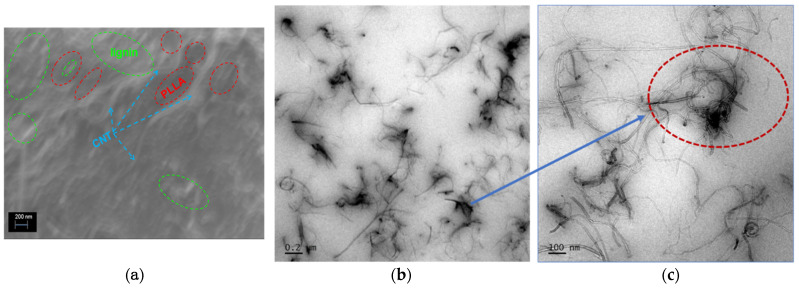
(**a**) FESEM image of PLLA_Lignin_MWCNT. PLLA matrix is indicated in red, and the areas containing lignin are shown in green. Blue arrows point at filament shapes, which are characteristic of MWCNT, (**b**,**c**) TEM images of PLLA_Lignin_MWCNT.

The main morphological features associated with the dispersion of lignin and nanofillers into the PLLA matrix are shown in Figure 1, based on electron-microscopy analyses on PLLA_Lignin_MWCNT nanocomposites. One can observe the successful incorporation of lignin and nanofiller, appearing as light domains and white filaments, respectively, in the dark-colored PLLA matrix of the FESEM image, which is confirmed by the TEM image. The TEM micrograph shows the presence of MWCNT distributed homogeneously over lignin exhibiting good interaction between them. Lignin exhibits a highly complex chemical structure, in which several chemical functions can contribute to interfacial interactions, not only by the formation of hydrogen bonds through hydroxyl groups, but also by favoring secondary van der Waals interactions via phenyl and carbonyl groups, with the latter being particularly compatible with PLLA ester groups [42].

**Figure 2:**
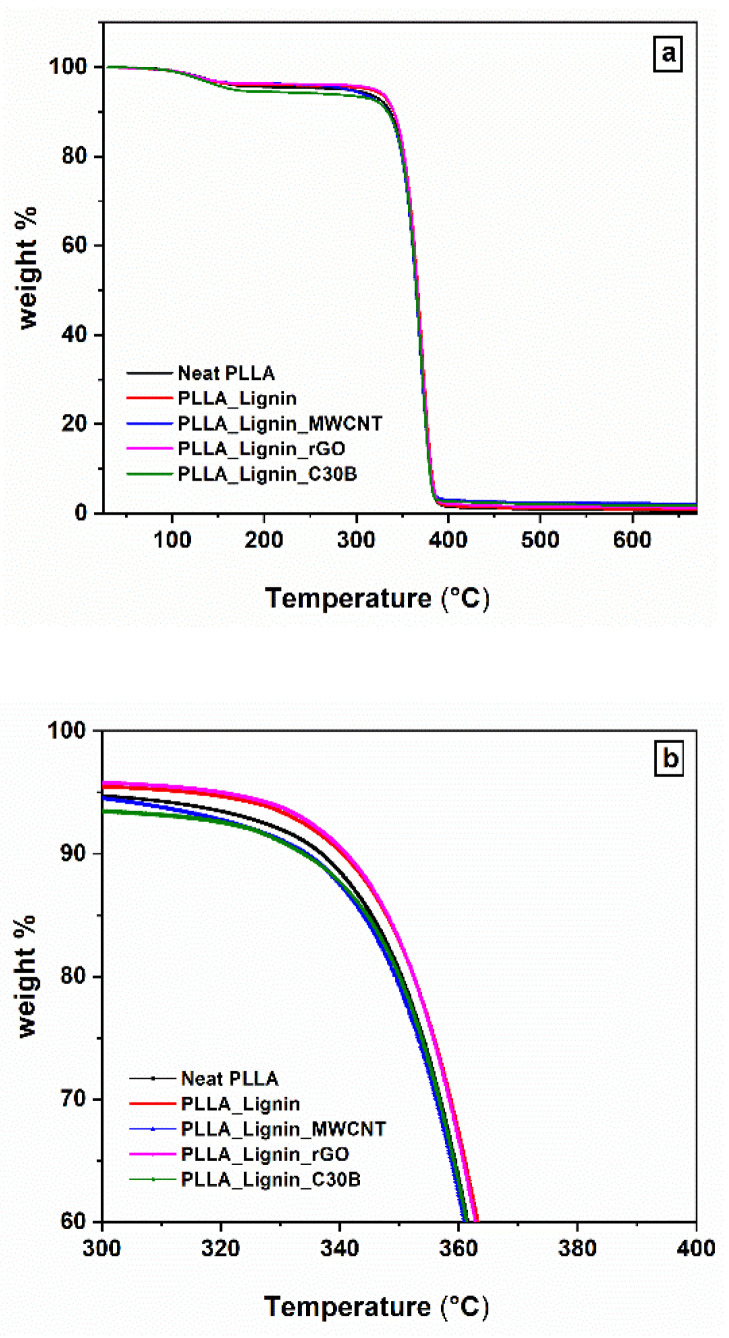
TGA profiles for neat PLLA, PLLA with additive, and nanocomposites. Influence of the filler choice: (**a**) global profile (**b**) focus on the onset of the main degradation step.

**Figure 3:**
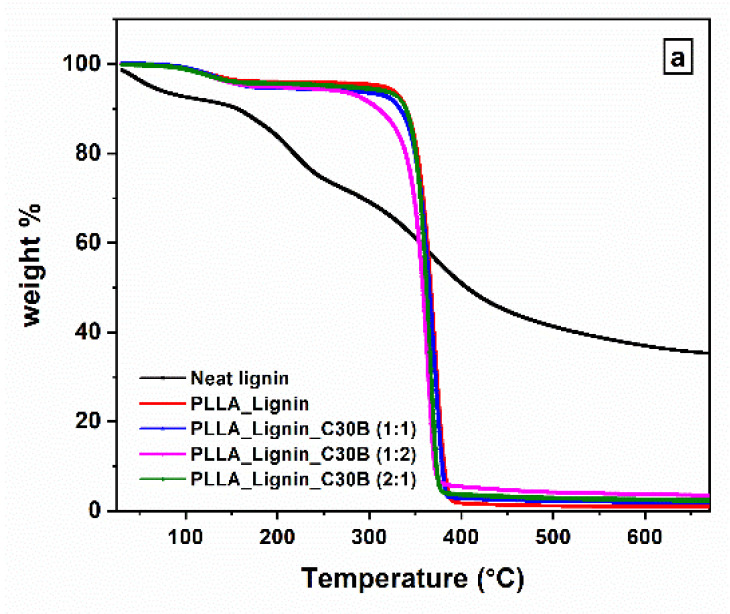

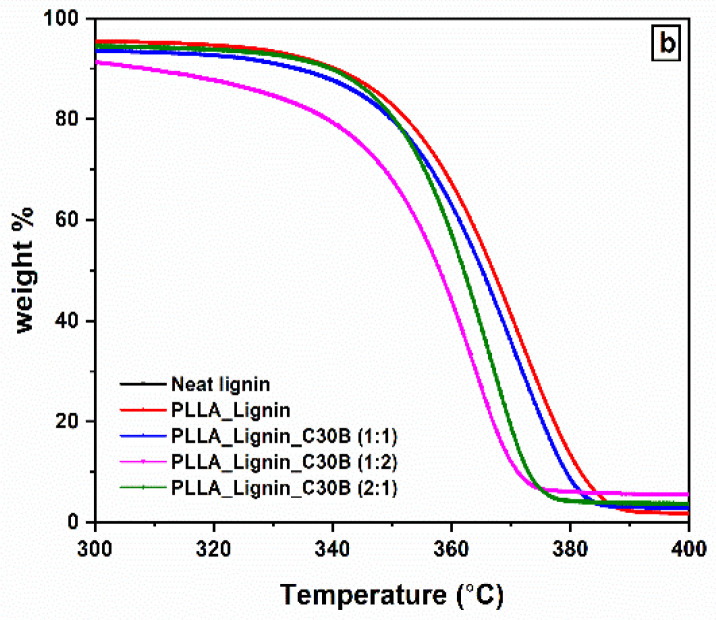
TGA profiles for PLLA with additive and nanocomposites. Influence of the lignin/filler ratio: (**a**) global profile (the TGA profile of lignin is added) (**b**) focus on the main degradation step.

Figure 2 and Figure 3 show the evolution of the weight percent as a function of temperature in PLLA_Lignin and hybrid nanocomposites, depending on the nanofiller nature (Figure 2) and on the lignin/nanofiller ratio (Figure 3). Data regarding neat PLLA and neat lignin were also added in Figure 2 and Figure 3, respectively. The degradation of neat lignin occurs in three stages, named the drying stage, the fast-degradation stage, and the slow-degradation stage by Ma et al. [43]. The degradation of PLA-based materials occurs in two steps: The weight loss corresponding to the temperature range 100–170 °C for all samples might be due to the presence of water, and the major weight loss observed in the range 280–395 °C might be due to the complete degradation of the matrix. Temperatures *T_w%_* associated with representative mass-loss percentage *w*% are given in Table 1, with *w* = {5; 10; 50; 90; 95}. 

The *T*_5%_ values are dispersed because of slight variations regarding the water content in the samples. *T*_10%_ seems to be the most significant temperature at which to compare the thermal stability among nanocomposites, since it corresponds to the beginning of the matrix-degradation step (Figure 2b and Figure 3b). First, the addition of lignin seems to slightly improve the thermal stability, as expected [44]. Nanocomposites for which the lignin/nanofiller ratio is (1:1) show small differences in their TGA profile. Some are consistent with previous results from the literature [45,46], e.g., rGO improves the thermal stability, whereas MWCNT induces the opposite effect. On the other hand, our results show accelerated degradation with the addition of C30B in contrast to the reported stabilizing effects [47]. The antagonist actions of both lignin and C30B are highlighted by modifying their ratio. *T*_10%_ shifts to higher values in PLLA_Lignin_C30B (2:1) but severely drops in PLLA_Lignin_C30B (1:2). 

*T*_50%_, *T*_90%_, and *T*_95%_ are globally homogenous among materials, with the exception of PLLA_Lignin_C30B (2:1) and PLLA_Lignin_C30B (1:2). Both exhibit lower values of *T*_50%_ and **T_90%_ compared to other systems, which is more surprising for PLLA_Lignin_C30B (2:1). We may assume that lignin, partly degraded, loses its stabilizing effect at these advanced stages of pyrolysis. *T*_95%_ is curiously high in PLLA_Lignin_C30B (1:2). However, this result might be influenced by the nanofiller content that does not degrade, which may hinder the release of gases. 

**Figure 4:**
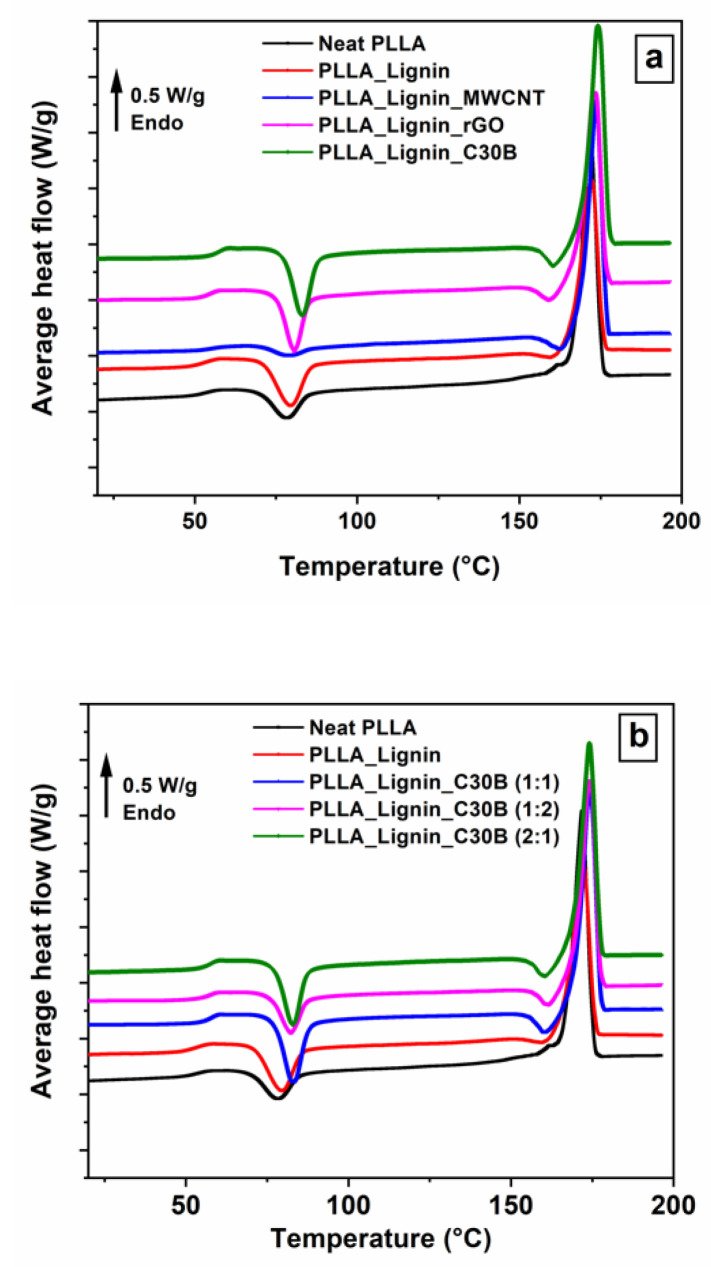
MT-DSC heat-only average heat flow signals: (**a**) influence of the filler choice; (**b**) influence of the lignin/filler ratio.

Figure 4 gives the evolution of the MT-DSC average heat flow as a function of the temperature for all the samples, consecutive to cooling from the melt at 50 K min^−^^1^. The classical behavior obtained for neat PLLA was observed as follows: a heat-flow step of around 55–60 °C, corresponding to the glass transition; an exothermic peak with a maximum at around 80 °C, corresponding to the cold crystallization; and an endothermic peak at around 170 °C, indicating the melting temperature. The addition of lignin brought about a remarkable change in the thermal behavior of the PLLA. Indeed, we observed that the amplitude of the heat-capacity step Δ*Cp* at the glass transition and the enthalpy of cold crystallization Δ*H_c_* were increased when the glass transition temperature *T_g_* shifted to lower temperatures. These changes are the signature of a greater amorphization during cooling. The degree of crystallinity *X_c_*, calculated according to Equation (2) and given in Table 1, confirms that the addition of lignin retards the PLLA crystallization.

(2) $$X_{C}=\frac{∆H_{f}-\Sigma ∆H_{c}\;}{∆H_{f}{}^{\circ}}$$

where Δ*H_f_* is the enthalpy of melting and $∆H_{f}{}^{\circ}$ is the enthalpy of melting of 100% crystalline PLLA, which is considered to be equal to 93 J/g [48]. Δ*H_c_* and Δ*H_f_* were normalized to the mass of PLLA. The same normalization was applied to Δ*Cp* (see Table 1). 

The results presented in Figure 4a and Table 1 highlight that only PLLA_Lignin_MWCNT crystallized more than neat PLLA under cooling from the melt. According to Hu et al. [46], carbon nanotubes are more efficient nucleating agents than rGO. Barrau et al. [49] proposed that the high specific surface of carbon nanotubes implies a large number of nucleation sites. Therefore, the crystallization rate, and consequently *X_c_,* can be adjusted according to the choice of the filler. The results obtained for PLLA_Lignin_C30B show that additional control can be managed by modifying the filler/lignin ratio. This is interesting for industrial production, for which the cooling rate is not easily adjustable. 

One can assume that lignin creates specific interactions with both the matrix and nanofillers, which could differ in nature, size, or content, depending on the nanofillers. The idea behind the calculation of the CRR size is to use the glass-transition calorimetric response as a fingerprint of the intermolecular architecture. To evaluate the impact of lignin and nanofillers properly, it is mandatory to investigate amorphous samples. Differences are observed regarding Δ*Cp*, *T_α_*, and Δ*T*, impacting the CRR size. The results are presented in Table 2 for all materials.

**Figure 5:**
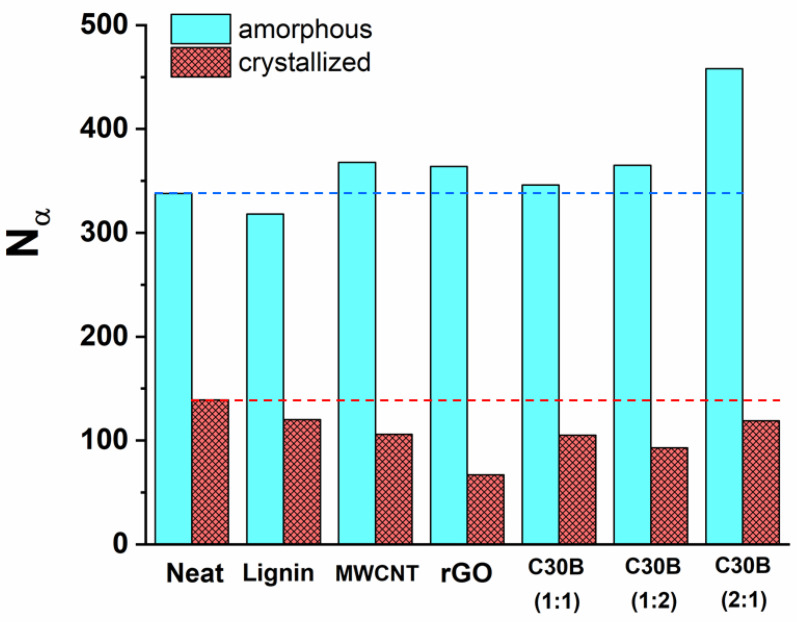
Number of relaxing units per CRR in amorphous and semi-crystalline PLLA and nanocomposites. Dashed lines are a guide, showing *N_α_* for amorphous neat PLLA.

Figure 5 and Table 2 show that the hybrid nanocomposites exhibit a slight increase of *N_α_* and Δ*Cp*. Classically reported effects of nanofillers on the calorimetric response at the glass transition are a decrease of Δ*Cp*, attributed to the existence of *X_RAF filler_* [35], and a decrease of *N_α_* [34], which is a consequence of mobility restrictions likely induced by *X_RAF filler_*; both effects are in contradiction with our results. Equation (3), in which Δ*Cp°* is Δ*Cp* of neat amorphous PLLA, is not valid in our case, as it leads to negative values for *X_RAF filler_*.

(3) $$X_{RAF\;filler}=\;1-\frac{∆Cp\;}{∆Cp{}^{\circ}}$$

**Figure 6:**
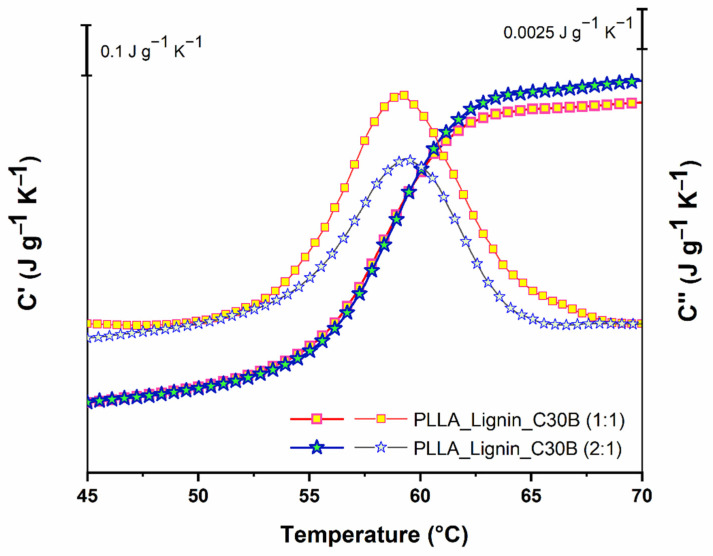
In-phase *C*′ and out-of-phase *C*″ components of C*, the complex heat capacity for PLLA_Lignin_C30B (1:1) and PLLA_Lignin_C30B (2:1) as a function of temperature from the MT-DSC heat-cool protocol: *C*′ appears as a step and *C*″ as a peak. The thick markers and lines are used to plot *C*′, whereas thin markers and lines refer to *C*″.

To explain the increase of Δ*Cp* and *N_α_*, we assumed a successful exfoliation [34], which favored intermolecular interactions without generating *X_RAF filler_*, i.e., *X_RAF filler_* was equal to zero. Recently, Szymoniak et al. also reported an increase of Δ*Cp* in epoxy-based nanocomposites [50], reaching an optimum before falling with the increase of the filler content, probably due to the competition existing between the creation of interactions and the mobility restrictions. According to our results, *N_α_* is equal to 458 in PLLA_Lignin_C30B (2:1). Figure 6 shows *C*′ and *C*″ signals for this system and PLLA_Lignin_C30B (1:1) for comparison. Such an increase in *N_α_* in comparison with neat PLLA exceeded our expectations. We assume that this characterizes the role of lignin as an interaction promoter in hybrid nanocomposites.

These considerations do not extend beyond amorphous systems. In semi-crystalline nanocomposites, the mobility criteria are strongly affected, and the response observed for amorphous systems is usually not reproduced. This is particularly obvious when high values of *X_RAF crystal_* are obtained, leading to a huge decrease in CRR size [34]. Therefore, we investigated the cooperativity in semi-crystalline nanocomposites annealed in conditions helping to grow *X_RAF crystal_* at a low temperature from the glassy state [51]. The results are given in Table 2. *X_RAF_*, i.e., the total amount of rigid amorphous fraction in the investigated material is calculated according to Equation (4). *X_RAF crystal_* is obtained by subtracting *X_RAF filler_* from *X_RAF_*. Because *X_RAF filler_* is null, *X_RAF crystal_* is simply equal to *X_RAF_*.

(4) $$X_{RAF}=X_{RAF\;crys}=1-\frac{∆Cp\;}{∆Cp{}^{\circ}\;}-X_{c}$$

First, *N_α_* decreased from about 340 to 140 by annealing neat PLLA, as expected. *X_c_* and *X_RAF crystal_* were similar between samples, but *N_α_* was the highest for neat PLLA. A comparison among PLLA_Lignin_C30B materials revealed that this decrease in the CRR size was not related to the lignin, but to the nanofiller. Finally, *N_α_* depends on the nature of the nanofiller. It reached the lowest value of 67 for PLLA_Lignin_rGO. Considering that *X_c_* and *X_RAF crystal_* are similar between samples, this could reasonably indicate that the crystalline morphology differs. It has already been reported that PLA-based nanocomposites containing rGO nanoflakes are characterized by very imperfect crystals in comparison to neat PLLA [45]. For equally high measurements of *X_c_* and *X_RAF crystal_*, a decrease in the long period, i.e., in the spacing between adjacent crystalline lamellae layers, will result in a more efficient propagation of the mobility restriction from the crystal to the mobile amorphous through the RAF. To summarize, the results obtained for amorphous nanocomposites confirm the role of lignin as a promoter of interfacial interactions because the calorimetric response is highly sensitive to intermolecular interactions. On the other hand, the microstructure and the morphology govern the glass-transition signature in semi-crystalline nanocomposites, which is consistent with previous observations [34,52]. 

The structural dependence of the CRR size is not systematically accompanied by similar variations in other relaxation parameters characteristic of the glass-transition dynamics. The kinetic fragility index, for example [53], which characterizes the degree of deviation from the Arrhenius-type temperature dependence of the relaxation time when approaching the glass transition during cooling, can show complex variations with cooperativity [54]. Hong et al. [55] separated the fragility into two terms, volume and energetic, with only the first being correlated with cooperativity. This volume term has been interpreted by Araujo et al. [56] as the contribution of interchain interactions to the relaxation motions. One may assume that the kinetic fragility increases, along with cooperativity, by adding lignin to PLLA nanocomposites, due to the increase of the volume term; however, further investigations are needed to validate this assumption. Indeed, various trends can be found in the literature regarding fragility variations with nanofiller inclusion [24,50,57,58,59], and it has been shown that cooperativity and fragility can evolve in opposition [60]. 

## 4. Conclusions

Calorimetric studies can provide information regarding both interactions and mobility restrictions at the filler/matrix interface of nanocomposites, which is helpful for completing the morphological diagnostic, often limited to the filler-dispersion evaluation. It emerged from our results that lignin is a promising additive for the design of PLLA-based nanocomposites. Even when added in low amounts, it promotes a homogeneous dispersion or exfoliation when creating weak interactions with both filler and matrix, and regularly increases the thermal stability. Interestingly, lignin is versatile regarding its impact on PLLA-crystallization kinetic, depending on the chosen added nanofiller, which is of tremendous relevance for the material design. Moreover, the possible association between lignin and several nanofillers, exhibiting different shape, size, and dimensionality, offers other ways of modulating the macroscopic properties, as observed from the difference in amorphous dynamics among semi-crystalline samples. It is worth noting that lignin is a macromolecule that can be abundantly extracted from biomass. Thus, this study might contribute to the valorization of lignin for sustainable development. 

## Figures and Tables

**Table 1:** TGA and MT-DSC heat-only extracted parameters.

	TGA					MT–DSC			
	Temperatures Corresponding to % of Weight Loss (°C)					Heat Capacity Step Δ*Cp* (J g^−1^ K^−1^), Glass-Transition Temperature *T_g_* (°C), Enthalpy of Cold–Crystallization Δ*H_c_* (J g^−1^), Degree of Crystallinity *X_c_* (%)			
	*T*_5%_	*T*_10%_	*T*_50%_	*T*_90%_	*T*_95%_	Δ*Cp*	*T_g_*	Δ*H_c_*	*X_c_* *
neat PLLA	289	337	365	380	383	0.21	53.5	16	41
PLLA_Lignin	315	340	367	381	385	0.42	52.5	33	18
PLLA_Lignin_MWCNT	291	334	365	380	383	0.14	54.0	10	43
PLLA_Lignin_rGO	321	341	366	381	385	0.38	55.0	31	23
PLLA_Lignin_C30B (1:1)	166	334	365	379	382	0.43	56.5	30	23
PLLA_Lignin_C30B (1:2)	205	309	358	371	437	0.38	56.5	23	28
PLLA_Lignin_C30B (2:1)	270	340	362	373	377	0.42	56.5	29	21

* Uncertainties regarding *X_c_* are ±2% from data reproducibility.

**Table 2:** Rigid amorphous fraction and crystalline-phase contents, as well as the characteristic amorphous-phase parameters extracted from the C’ and C’’ signals (Δ*Cp*, *T_α_*, and Δ*T*) that were used for the CRR-size calculation.

	Amorphous				Semi-crystalline					
	*T_α_* (°C)	Δ*T* (°C)	Δ*Cp* (J g^−1^ K^−1^)	*N_α_*	*T_α_* (°C)	Δ*T* (°C)	Δ*Cp* (J g^−1^ K^−1^)	*X_c_* * (%)	*X_RAF crystal_* ** (%)	*N_α_*
neat PLLA	58.5	2.70	0.54	338	67.0	4.3	0.13	32	45	139
PLLA_Lignin	58.2	2.75	0.53	318	64.0	4.6	0.15	33	39	120
PLLA_Lignin_MWCNT	58.8	2.63	0.57	368	64.6	5.0	0.17	30	39	106
PLLA_Lignin_rGO	58.4	2.70	0.60	364	65.0	6.4	0.16	33	40	67
PLLA_Lignin_C30B (1:1)	59.2	2.68	0.54	346	67.2	4.9	0.18	31	35	105
PLLA_Lignin_C30B (1:2)	59.2	2.63	0.56	365	67.2	5.35	0.16	31	39	93
PLLA_Lignin_C30B (2:1)	59.6	2.40	0.58	458	65.6	4.8	0.19	29	40	119

* Uncertainties regarding *X_c_* are ±2% from data reproducibility. ** Uncertainties regarding *X_RAF crystal_* are ±5% from data reproducibility.

## Data Availability

The data presented in this study are available upon request from the corresponding author.
